# Aneurysms of Peripancreatic Arterial Arcades Coexisting with Celiac Trunk Stenosis or Occlusion: Single Institution Experience

**DOI:** 10.1155/2017/1645013

**Published:** 2017-02-13

**Authors:** Robert Antoniak, Laretta Grabowska-Derlatka, Ireneusz Nawrot, Andrzej Cieszanowski, Olgierd Rowiński

**Affiliations:** ^1^2nd Department of Radiology, Medical University of Warsaw, Banacha 1a St., 02-097 Warsaw, Poland; ^2^Department of General, Vascular, and Transplantation Surgery, Medical University of Warsaw, Banacha 1a St., 02-097 Warsaw, Poland

## Abstract

*Introduction*. True aneurysms of peripancreatic arterial arcades (PAAAs) are rare. Most of them coexist with celiac axis stenosis/occlusion due to median arcuate ligament (MAL) compression or atherosclerosis. The aim of this study was to evaluate the cause of celiac axis lesion and characterize the anatomy of the aneurysms. These findings may have important management implications.* Material and Methods*. A retrospective analysis of 15 patients with true PAAAs was performed. The diagnosis was established by contrast-enhanced CT, using a 64-MDCT scanner. We evaluated the most probable cause of celiac axis lesion. Aneurysms were characterized by their number, location, size, and morphology. Location of the aneurysms was classified either as pancreaticoduodenal arteries (PDA) or as dorsal pancreatic arteries (DPA) as they may represent different collateral pathways between superior mesenteric artery and celiac trunk.* Results*. A total of 32 true PAAAs were identified. Celiac trunk was occluded in 12 patients and critically narrowed in 3 patients. Celiac axis lesion was categorized as secondary to MAL compression in 14 cases and due to atherosclerosis in 1 case. The most common location of the aneurysms was inferior pancreaticoduodenal arteries. Only in 1 case aneurysms involved both PDA and DPA.* Conclusions*. Coexistence of PAAAs with celiac axis compression as well as involvement of either PDAs or DPAs has important therapeutic implications. The uninvolved collateral pathway may be sufficient to preserve effective circulation in celiac trunk branches in case of resection or embolization of the aneurysms. However, further studies are crucial to confirm our findings.

## 1. Introduction

Aneurysms of peripancreatic arterial arcades (PAAAs) are uncommon as they account for about 2% of all visceral aneurysms. The distinction between true and false aneurysms is challenging. History of acute pancreatitis, pancreatic trauma, or surgery points towards false aneurysms. Lack of these risk factors favors true aneurysms, especially when celiac axis stenosis/occlusion coexists. Celiac axis lesion is the most common cause of true PAAAs formation. However, many other etiologies have been described, including vasculitis [1], fibrodysplasia [2], and congenital collagen disorders [3]. True PAAAs are rare and therefore literature covering this entity is scarce. Predominantly, case reports and studies with only limited number of patient cases are being published. Until August 2011 only 93 cases of true PAAAs were reported [4]. The aim of this study was to elucidate the features of PAAAs with reference to the strong associations with celiac axis lesions. The main consideration points included etiology of celiac axis lesion as well as location of the aneurysms on different collateral pathways between superior mesenteric artery and celiac axis.

## 2. Material and Methods

A retrospective review of 15 patients with PAAAs hospitalized in our institution was performed. The mean patients' age was 58 years, ranging from 31 to 84. None of the patients had a history of acute pancreatitis, pancreatic trauma, or surgery. All but one patient presented with nonspecific abdominal complaints. Only one patient suffered postprandial epigastric pain and at the same time had a diagnosis of* H*.* pylori* infection. In all cases the diagnosis was made incidentally. The diagnosis of PAAAs was established by contrast-enhanced CT in all cases. All studies were performed using a 64-MDCT (Light Speed, GE), which was used according to protocol: arterial-phase CT; slice thickness 1.2 mm. The angio-CT examination was performed during intravenous administration of a bolus of contrast agent (low-osmolarity contrast 80 ml; injection rate 5 ml/s). Secondary reconstructions were obtained with the application of volume rendering (VR) and maximum intensity projection (MIP) algorithms. For the purpose of this analysis all studies were reevaluated by two radiologists.

**Figure 1:**
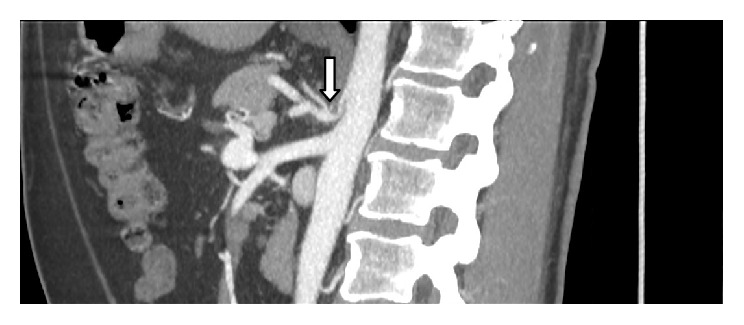
Thickened median arcuate ligament (MAL) and typical celiac axis narrowing with a hooked appearance pointed towards compression syndrome (arrow).

We paid special attention to the patency of celiac axis and superior mesenteric artery. The most likely cause of their stenosis/occlusion was noted. Absence of atherosclerotic plaques, thickened MAL, and typical celiac axis narrowing with a hooked appearance pointed towards compression syndrome (Figure 1). Thickness of MAL was measured and divided into severe or nonsevere with a threshold value of 4 mm (Table 1).

Presence of a significant atherosclerotic plaque in the celiac trunk ostium favored the atherosclerotic etiology. We characterized the aneurysms by their number, location, size, and morphology (Table 2). We separated aneurysms involving PDAs and GDA from that involving DPA as these may represent different collateral pathways between superior mesenteric artery and celiac axis. Despite the small study group we tried to find any relationship between patients' age or sex and aneurysms' number or size. The hospital's investigation review board approved this retrospective study and waived the need for individual patient consent.

## 3. Results

**Figure 2:**
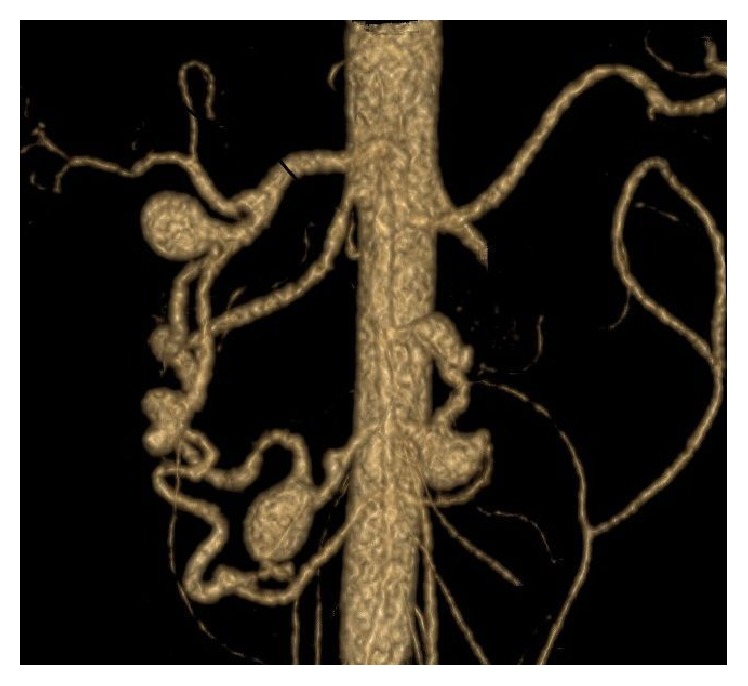
True PAAAs.

**Figure 3:**
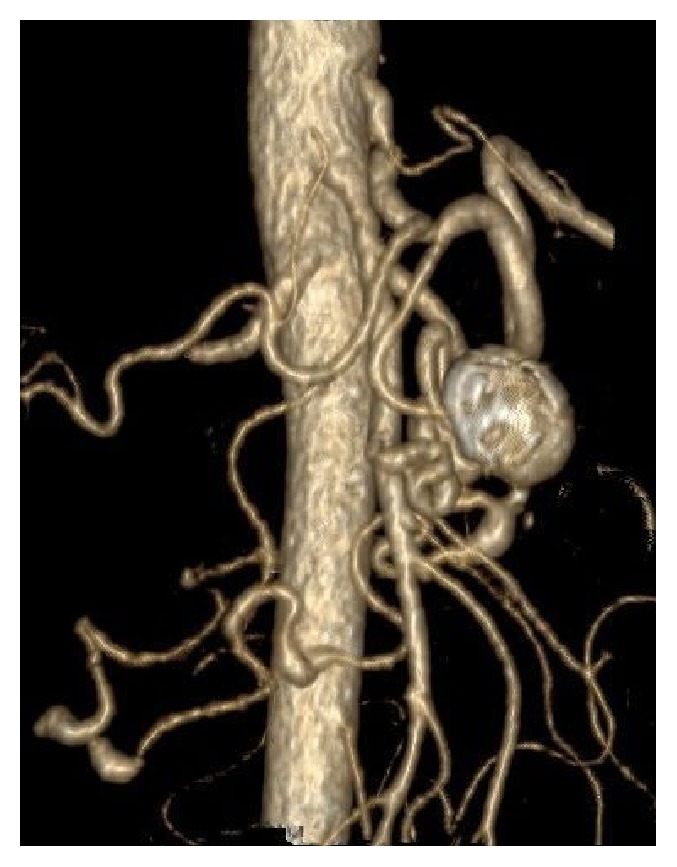
True PAAA.

In our review, a total of 29 true PAAAs were identified (Figures 2 and 3). The aneurysms were multiple in eight patients, with a maximum of 6 aneurysms in one patient. Seven patients had a solitary aneurysm. Vast majority of the aneurysms were saccular (27 of 29, 93%). Their most common locations were PDAs, especially inferior pancreaticoduodenal arteries (13 cases). Additionally, 3 aneurysms originated on GDA. DPA or its branches were involved in 7 cases. Size of the aneurysms ranged from 3/3/3 mm to 50/51/53 mm (median size 13/12/12 mm, tr/ap/cc). The detailed data is summarized in Table 2.

**Figure 4:**
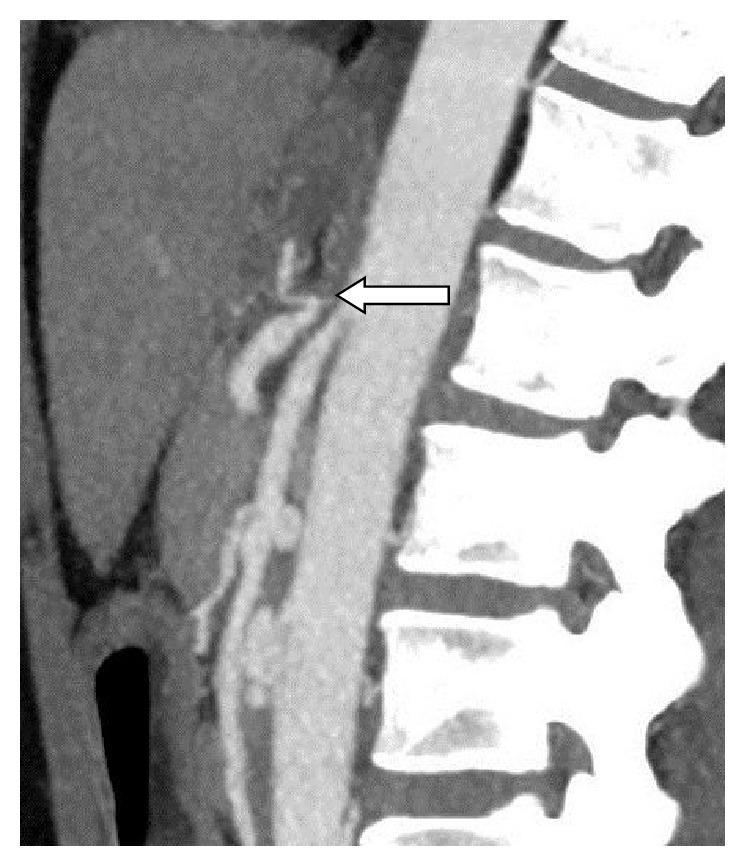
Superior mesenteric artery stenosis (arrow) as a consequence of MAL compression.

Celiac trunk occlusion was identified in 12 patients and it is critical narrowing in 3 patients. Thickness of MAL was classified as severe in 10 and nonsevere in 5 cases. Fourteen patients had a proximal celiac trunk narrowing with an appearance characteristic for MAL compression or occlusion with prominent MAL. Atherosclerotic plaques in celiac axis ostium were found in 3 patients. In two of them the plaque was minimal. Only one patient had a significant ostial plaque of an occluded celiac trunk. Therefore, the celiac axis lesion was classified as secondary to MAL compression in 14 cases and atherosclerosis in 1 case. One patient had a concomitant superior mesenteric artery stenosis as a consequence of MAL compression (Figure 4).

## 4. Discussion

True PAAAs are rare and not well characterized. They are discovered usually in 6th-7th decade of life, with no sex predominance [5]. The uncoincidental relationship between PAAAs and celiac axis stenosis/occlusion is well known and was first described by Sutton and Lawton in 1973. It is believed that increased blood flow through the collateral circulation to main hepatic artery via PDAs and DPA results in enlargement of these vessels. The overload of the arteries produces local tears in their walls and subsequently leads to aneurysm formation [5–8]. This theory has recently been supported using flow-sensitive four-dimensional magnetic resonance imaging [9]. In the literature, there are found cases of PAAAs with celiac axis lesion due to either atherosclerosis or compression by MAL. Previous reviews suggested that 35% of GDA and as many as 62% of PDA aneurysms are ruptured at presentation [8, 10–13]. However, with the widespread use of US and CT studies, the increasing number of aneurysms is diagnosed in asymptomatic patients. The data concerning formation and growth rate of the aneurysms is very little. Rupture of PAAAs remains unpredictable. Neither size and multiplicity of the aneurysms nor age of the patients correlates with the risk of rupture [14]. Therefore, management strategy of unruptured PAAAs is controversial.

We performed a retrospective review of 15 patients with PAAAs. To the best of our knowledge, this is the largest group of PAAA aneurysm cases associated with celiac axis lesions. All patients had celiac axis occlusion or critical stenosis. In 14 patients the celiac trunk lesion was secondary to compression by MAL, while in only 1 significant, proximal celiac trunk atherosclerosis was present. Furthermore, we presume that atherosclerotic plaques in celiac axis ostium do not exclude the preexisting compression. In 3 cases of celiac axis compression the vessel was critically narrowed. It is worth noticing that CT scans are usually obtained on inspiration when the compression is partially released. Therefore, we can suspect that in these 3 cases celiac trunk could have been occluded on expiration.

Only one of our patients presented with epigastric pain, which could be attributed to concomitant* H*.* pylori* infection. Other patients were asymptomatic. It confirms the difficulty in diagnosing the aneurysms and indicates that majority of them remain undiagnosed [15].

The delineation of separate collateral pathways between superior mesenteric artery and celiac axis is frequently impossible on CT and the exact anatomy can be obtained only with angiography. PDAs and DPA may represent different pathways. PAAAs in our study group originated predominantly on PDAs. Interestingly, only in one case both pathways were involved. We have not found any significant relationship between patients' age or sex and number or size of the aneurysms. Each woman had 2,3 aneurysms, while each had man −1,3 aneurysms; however the study group was too small to draw conclusion on sex predilection of PAAAs. Lack of relationship between patients' age and size of the aneurysms may indicate that the aneurysms remain stable over time. The fact that there are no known cases of PAAAs recurrence in case of celiac axis stenosis/occlusion seems to support this theory. The largest retrospective review of PAAAs followed without treatment included 5 patients with a total of 8 aneurysms [16]. In this series five aneurysms remained stable and three enlarged. Of these three aneurysms two increased their size by only 1 mm in 22-month follow-up. Further long-term follow-up studies are necessary to evaluate the natural history of PAAAs.

## 5. Conclusion

All 15 patients with PAAAs in our series had a coexisting celiac axis occlusion or critical stenosis. Etiology of celiac axis lesion is often difficult to characterize as both compression and atherosclerosis may coexist. In our series celiac axis lesion was most likely due to MAL compression in all but one case. The aneurysms involved predominantly PDAs. They originated on both PDAs and DPA in only one case. No relationship between aneurysms' size and patients' age may indirectly indicate stability of the aneurysms over time. All this features have important implications on PAAAs management. We have to emphasize that our study group was too small to draw any definite conclusions and further studies are needed to confirm our hypotheses.

## Figures and Tables

**Table 1:** Number, size, and localization of PAAAs.

Patient	Celiac axis (CA) occlusion/stenosis	Atherosclerosis (diffuse/CA ostium)		Median arcuate ligament thickness	Most likely cause of celiac axis lesion
1	Occlusion	NS	—	NS	Compression
2	Occlusion	NS	—	NS	Compression
3	Occlusion	—	—	S	Compression
4	Occlusion	NS	S	NS	Atherosclerosis
5	Stenosis	—	—	NS	Compression
6	Stenosis	—	—	S	Compression
7	Occlusion	NS	—	S	Compression
8	Occlusion	—	—	S	Compression
9	Stenosis	—	—	S	Compression
10	Occlusion	NS	NS	S	Compression
11	Occlusion	NS	NS	S	Compression
12	Occlusion	—	—	S	Compression
13	Occlusion	—	—	S	Compression
14	Occlusion	—	—	NS	Compression
15	Occlusion	NS	—	S	Compression

S: severe; NS: nonsevere.

**Table 2:** The cause and degree of celiac axis narrowing.

Patient	Number	Location	Size (mm) ml/ap/cc	Morphology
1	2	A. gastroduodenalis A. pancreaticoduodenalis sup. ant.	50/51/53 9/9/8	Saccular Saccular

2	2	A. gastroduodenalis A. pancreaticoduodenalis inf.	5/6/4 3/3/3	Saccular Saccular

3	6	A. gastroduodenalis A. pancreaticoduodenalis sup. ant. A. pancreaticoduodenalis inf. post. A. pancreaticoduodenalis inf. A. pancreaticoduodenalis sup. post. A. pancreaticoduodenalis inf.	16/15/15 8/7/13 14/14/17 14/14/11 7/6/16 8/8/21	Saccular Saccular Saccular Saccular Fusiform Fusiform

4	1	A. pancreaticoduodenalis inf. ant.	4/5/5	Saccular

5	1	A. pancreaticoduodenalis inf.	14/16/16	Saccular

6	1	A. pancreatica dors.	42/35/35	Saccular

7	2	A. hepatica dex. + a. pancreaticoduodenalis inf. ant. A. pancreaticoduodenalis inf. post.	14/22/18 9/9/9	Saccular Saccular

8	2	A. pancreaticoduodenalis inf. post. A. pancreaticoduodenalis inf. ant.	19/19/23 15/13/11	Saccular Saccular

9	1	A. pancreatica dors.	13/12/12	Saccular

10	1	A. pancreatica dors.	15/14/14	Saccular

11	1	A. pancreatica dors.	4/4/4	Saccular

12	2	A. pancreaticoduodenalis inf. A. pancreaticoduodenalis sup. ant.	18/27/31 14/15/9	Saccular Saccular

13	4	A. pancreatica dors. A. pancreatica dors. A. pancreaticoduodenalis inf. A. pancreaticoduodenalis sup. ant.	19/15/21 8/11/9 5/4/4 4/4/4	Saccular Saccular Saccular Saccular

14	1	A. pancreatica dors.	9/8/11	Saccular

15	2	A. pancreaticoduodenalis inf. A. pancreaticoduodenalis sup. post.	27/23/24 3/3/3	Saccular Saccular
